# The Effects of Iron-Bearing Minerals on the Community Diversity and Physiological Activity of Prokaryotic Microorganisms in Pit Mud Used for Strong-Flavor *baijiu* Production

**DOI:** 10.3390/foods14111883

**Published:** 2025-05-26

**Authors:** Kairui Jiao, Bo Deng, Ping Song, Liwei Wang, Bin Lian

**Affiliations:** 1 School of Life Sciences, Nanjing Normal University, Nanjing 210023, China; jkr08061999@163.com; 2 National Engineering Research Center of Solid State Brewing, Luzhou 646000, China; dengbo@lzlj.com; 3 School of Food Science and Pharmaceutical Engineering, Nanjing Normal University, Nanjing 210023, China; songping@njnu.edu.cn (P.S.); 18900505471@163.com (L.W.); 4 College of Marine Science and Engineering, Nanjing Normal University, Nanjing 210023, China

**Keywords:** pit mud, crystalline iron minerals, amorphous iron (hydr)oxides, microbial diversity, strong-flavor *baijiu*

## Abstract

The quality of strong-flavor *baijiu* largely depends on the physicochemical properties and prokaryotic microbial activities of pit mud. However, the impact of the iron-bearing minerals in pit mud on its prokaryotic microbial communities remains unknown. This study examined the differences in the prokaryotic communities between 2-year, 40-year, and 100-year pit mud and yellow soil (the raw material for pit mud), as well as the impacts of environmental factors, particularly iron-bearing minerals, on the structure and diversity of these prokaryotic communities. The results indicated that there were significant differences in the composition of prokaryotic microorganisms between yellow soil and pit mud. As the fermentation pit aged, the relative abundance of dominant fermentation bacteria (including *Petrimonas*, *Syntrophomonas*, *Clostridium*, etc.) and hydrogenotrophic methanogens in the pit mud increased. The relative abundance of *Lactobacillus* in the 2-year pit mud was low (0.33%). Under laboratory conditions, goethite (a typical crystalline iron mineral, denoted as Fe_c_) reduced the physiological and metabolic activity of *Lacticaseibacillus paracasei* JN01 in a concentration-dependent manner. The results of the physicochemical analysis showed that the contents of total iron (TFe) and Fe_c_ significantly decreased, while the contents of Fe(II) and amorphous iron (hydr)oxides (Fe_o_) significantly increased with an increasing fermentation pit age. TFe and Fe_c_ were significantly negatively correlated with both the Chao1 and Shannon indexes and functional microorganisms such as *Clostridium_sensu_stricto_12*, *Sedimentibacter*, and hydrogenotrophic methanogens. The current results contribute to our understanding of the aging process of pit mud from the perspective of the interaction between iron-bearing minerals and prokaryotic microorganisms.

## 1. Introduction

Strong-flavor *baijiu*, known for its robust flavor profile, fermentation-pit-like aroma, and harmonious taste, has earned global recognition and acclaim [1,2]. Luzhou Laojiao liquor, produced by Luzhou Laojiao Company located in Luzhou city in China, is an outstanding representative of strong-flavor *baijiu*, characterized by its use of a lutaceous fermentation pit for solid-state anaerobic fermentation. The underground fermentation pit’s walls and base are encased in a layer of pit mud, formulated of superior yellow soil, yellow water, vinasse, and wheat bran [3,4]. Long-term production practice has proven that the longer the lifespan of a fermentation pit, the superior the quality of its pit mud, consequently leading to a more intense aroma of strong-flavor *baijiu* [5].

The prokaryotic microbial community in pit mud plays an indispensable role in the fermentation of strong-flavor *baijiu* [6]. *Lactobacillus* typically dominates in young pit mud, while the relative abundance of functional microorganisms such as *Sedimentibacter*, *Petrimonas*, and hydrogenotrophic methanogens increases with the age of the fermentation pit [7,8]. The physicochemical environment of pit mud significantly affects the structure and diversity of its prokaryotic communities [4]. Numerous reports have explored the intricate relationship between the physicochemical properties of pit mud and its diverse microbial communities. Lactic acid, pH, and soluble Ca^2+^ were identified as the three predominant environmental factors that influence the structure of microbial communities in pit mud [7]. The relationship between physicochemical factors and microbial communities has been investigated in both new pit mud samples (5, 7, and 10 years old) and old ones (30 and 50 years old), revealing significant effects of key factors such as lactic acid, pH, caproic acid, etc., on bacterial community structure [4]. Pit mud samples from three regions in Sichuan Province, China, with varying fermentation pit ages (2, 3, 15, 20, and 50 years) were studied, and it was discovered that NH_4+_-N, available phosphorus, and moisture content (MC) were the primary driving forces behind bacterial community succession [9]. In general, existing studies indicate that the physicochemical environment and the material composition of pit mud exert a profound effect on the characteristics of microbial communities [4,7]. Nevertheless, these studies have not adequately focused on monitoring alterations in key mineral components and related elements during the aging process of pit mud.

Omnipresent minerals constitute the skeleton structure of pit mud [10]. The intricate interplay between microorganisms and minerals gives rise to a harmonious whole [11,12,13]. Hitherto, knowledge regarding the changes in minerals during the aging process of pit mud, as well as the impact of minerals on the prokaryotic microbial communities in pit mud, is still in its infancy. In a preliminary exploration, it was observed that as the age of the fermentation pit increased, the total iron (TFe), free iron minerals (Fe_d_), and crystalline iron minerals (Fe_c_) in the pit mud decreased significantly, while its Fe(II) and amorphous iron (hydr)oxide (Fe_o_) content increased remarkably [10]. However, this study did not assess the impact of iron-bearing minerals on the physiological activity and community structure of the prokaryotic microorganisms in pit mud. It is universally acknowledged that iron-bearing minerals can influence the community structure and physiological activity of soil microorganisms through a series of biological, physical, and chemical mechanisms [14,15]. For instance, acicular goethite (a typical representative of Fe_c_) can pierce the cell wall of *Bacillus subtilis* and reduce its survival rate [16]. The Fe(II) dissolved from iron-bearing minerals is oxidized into toxic Fe(III) precipitates after entering bacterial cells, which affects bacterial activity [17]. The addition of ferrihydrite (a kind of Fe_o_) altered the relative abundance of hydrogenotrophic methanogens in sediment [18]. The α and β diversity of the bacterial communities in the rhizosphere of maize was significantly altered after goethite treatment [19]. Collectively, iron-bearing minerals have a profound impact on the physiological activity and community-shaping of soil microorganisms [20,21]. Unfortunately, the influence of iron-bearing minerals on the physiological activity of prokaryotic microorganisms in pit mud and in-depth dissection of their relationship with prokaryotic microbial communities have been largely neglected.

To bridge this knowledge gap, the differences in the physicochemical properties and prokaryotic community structures between 2-year, 40-year, and 100-year pit mud and yellow soil (the raw material for pit mud) in Luzhou Laojiao distillery were evaluated. Furthermore, the correlation between physicochemical indexes, particularly the iron-bearing minerals, and the diversity of prokaryotic microbial communities in pit mud was also investigated. This study provides new insight into the aging process of pit mud from the perspective of the interactions between iron-bearing minerals and prokaryotic microorganisms.

## 2. Materials and Methods

### 2.1. Sample Collection

Samples were collected from Luzhou Laojiao distillery (105°29′26″ E, 28°53′51″ N), Luzhou city, Sichuan Province, China. Four groups of samples were collected, including yellow soil (HT) and pit mud aged for 2 years (PM2), 40 years (PM40), and 100 years (PM100), respectively. The sampling sites for pit mud were positioned at the diagonal intersections of the four walls and at the bottom, so five samples of pit mud were collected from each fermentation pit. To address the potential heterogeneity of the five samples of pit mud, they were homogenized, respectively, and then equally divided into four parallel samples. The four samples of yellow soil were collected from Wudu Brook by Luzhou Laojiao distillery. Each sample weighed approximately 200 g. The aforementioned samples were collected using aseptic collection bags, transferred into anaerobic-gas-generating bags to maintain anaerobic conditions, and then stored in a freezer set at −20 °C for future use.

### 2.2. Determination of the Physicochemical Properties of the Samples

The samples’ pH values were measured using a pH meter (PHSJ-4F, Shanghai Lei Ci, China) using a 1:2.5 soil-to-water ratio (mass/volume), following the Chinese National Standard NY/T 1377-2007 [10]. The samples’ moisture content (MC) was ascertained through a gravimetric analysis after the samples had been heated in an oven to 105 °C until they achieved a constant mass, in compliance with the Chinese National Standard LY/T 1213-1999 [10]. The levels of Fe(II) in the fresh samples were assessed employing a phenazine technique [22]. The TFe content in the samples was determined after the samples were digested with HNO_3_-HF-HClO_4_ mixed acid [23]. The Fe(III) content of each sample was expressed as the difference between the TFe content and the Fe(II) content [24]. Fe_d_ was isolated through extraction with a solution of sodium disulfite–sodium citrate–sodium bicarbonate at a concentration of 0.1 mol/L [25]. Fe_o_ was obtained by extracting it using an oxalate–ammonium oxalate solution at a concentration of 0.2 mol/L with the pH adjusted to 3 [26]. The Fe_c_ content was expressed as the difference between the Fe_d_ content and the Fe_o_ content [27]. The concentration of iron ions in the extract was measured using a flame atomic absorption spectrometer (AA-6300C, Shimadzu, Kyoto, Japan).

### 2.3. High-Throughput Sequencing and Isolation of the Lacticaseibacillus Strain

The total DNA in each sample was extracted using the soil DNA extraction kit (TianGen). The genomic DNA in the V3-V4 hypervariable region of 16S rDNA was amplified using the primers 343F-5′-TACGGRAGGCAGCAG-3′ and 798R-5′-AGGGTATCTAATCCT-3′. High-throughput sequencing was conducted on the NovaSeq6000 PE250 sequencing platform from Novogene in China. To ensure the readability of the sequencing data, the following procedures were carried out: (1) Data splitting: According to the sequence of the barcode and the PCR-amplified primers, each sample’s data were separated from the original data using Python (Version 3.6.13). (2) Double-ended data splicing: The barcode and primer sequences were truncated, and the reads for each sample were spliced using FLASH (Version 1.2.11). The spliced sequences were raw tags. (3) Chimera removal: Effective tags were obtained after chimera sequence removal using vsearch (Version 2.16.0). (4) The DADA2 module in QIIME2 (Version 2020.02) software was used to de-noise and de-weight effective tags, and amplicon sequence variants (ASVs) and a feature list were obtained. The Classify-sklearn algorithm and the naive Bayes classifier in QIIME2 software were used to annotate the species for each ASV. The 16S rDNA gene database was Silva 138.1 (https://www.arb-silva.de/documentation/release-1382/, accessed on 8 June 2023). To assess the differences in the richness and diversity of the prokaryotic communities within each sample, the α diversity indexes (observed ASVs and Chao1, Shannon, and Simpson indexes) were calculated using QIIME2 software, and the significance of the difference between groups was analyzed using Tukey’s test (*p* < 0.05) [4]. A principal coordinate analysis (PCoA, based on the Bray–Curtis distances and the ADONIS test) was used to analyze the differences in the prokaryotic community structures in different samples [9]. A canonical correspondence analysis (CCA) was utilized to calculate the correlation between the prokaryotic communities and environmental factors, and the envfit function was used to test the significance of each environmental factor. A Spearman’s rank correlation analysis was used to study the correlation between (1) environmental factors and prokaryotic communities at the genus level and (2) the correlation between environmental factors and the α diversity indexes of the prokaryotic communities in pit mud. FAPROTAX software (Version 1.2.4) was used to predict the function of the prokaryotic communities, and Tukey’s test was used to determine the significance of functional differences between groups. The linear discriminant analysis (LDA) effect size was used to identify species with significant differences between groups at the phylum and genus levels (called biomarkers). Species with LDA values greater than four were statistically different between groups. The sequencing data were submitted to the Sequence Read Archive (SRA) of the National Center for Biotechnology Information (NCBI) database under BioProject PRJNA1100219.

*Lacticaseibacillus* strains were screened using De Man, Rogosa, and Sharpe (MRS) medium [28]. The components of the MRS medium (g/L) were as follows: 20.0 glucose, 10.0 peptone, 5.0 beef extract, 4.0 yeast extract, 5.0 CH_3_COONa, 2.0 C_6_H_14_N_2_O_7_, 2.0 K_2_HPO_4_, 0.2 MgSO_4_•7H_2_O, 0.05 MnSO_4_•H_2_O, 1.0 Tween 80, and 15.0 agar. The medium without agar was liquid MRS medium. All media were at natural pHs of around 6.5. All of the media were sterilized at 115 °C for 30 min. A proportion of 10.0 g of pit mud was added to 100 mL of sterile water and homogenized. Then, 100 μL of the pit mud suspension was diluted 1000 times and coated onto the MRS medium, which was then incubated anaerobically in an incubator at 37 °C for 48 h. Suspected strains of *Lacticaseibacillus* were screened according to the morphology of the colonies (round, white on the front, and yellow on the back), the catalase test (negative), and Gram staining results (positive) [29]. Suspected strains were sent to Shanghai Sangon Biotech Co., Ltd. (Shanghai, China) for 16s rDNA strain identification. The strain sequencing results were compared with the data in the NCBI database according to the Basic Local Alignment Search Tool (BLAST) program. The strains with the highest homology with the tested strains were selected, and then a phylogenetic tree was constructed using the neighbor-joining method in MEGA 11 software.

### 2.4. Preparation and Characterization of Goethite

Goethite can be found in yellow soil and pit mud of various fermentation pit ages [10] and is often considered representative of crystalline iron minerals [30]. Therefore, goethite was prepared as follows [31]: KOH solution (200 mL, 2.5 mol/L) was added to Fe(NO_3_)_3_•9H_2_O solution (825 mL, 0.15 mol/L) and thoroughly mixed. After aging the suspension in an incubator at 60 °C for 48 h, the precipitation of goethite was collected, and the saline impurities on the surface of the goethite were removed using ultra-pure water. The goethite was dried (65 °C, 24 h) and ground to pass through a 100-mesh sieve. The crystal structure was analyzed using X-ray diffraction (XRD-BTX using Co-Kα radiation, Olympus, Waltham, MA, USA). Scanning electron microscopy (SEM, Gemini Sigma 300, Zeiss, Germany) was adopted to characterize the morphology of the goethite.

### 2.5. The Effects of Goethite on the Growth and Metabolism of Lacticaseibacillus paracasei JN01

#### 2.5.1. The Static Culture Experiment

Amounts of 0.03 g and 0.09 g of goethite were placed into 50 mL flat-bottomed centrifuge tubes containing 29.4 mL of the liquid MRS medium. The centrifuge tubes were sterilized (115 °C, 30 min) and then inoculated with 0.6 mL of strain JN01 seed solution (about 8 × 10^8^ cfu/mL). Therefore, the concentrations of goethite in the experimental group were 1 g/L and 3 g/L, respectively. A control group (CK) was prepared using 0.6 mL of the seed solution and 29.4 mL of the liquid MRS medium. Control group 1 (CK1) contained 0.03 g of goethite mixed with 30 mL of liquid MRS medium. Control group 2 (CK2) contained 0.09 g of goethite mixed with 30 mL of liquid MRS medium. Three replicates were established for each group. The centrifuge tube was sealed with a matching lid and placed in an incubator at 37 °C for static culture. Samples were collected at 24 h, 48 h, 72 h, and 96 h to determine their relevant physicochemical indexes.

#### 2.5.2. Determination of Physicochemical Indexes

In order to determine the relevant physicochemical indexes of the culture solution in the centrifuge tube, 1 mL of the culture solution was collected at the 25 mL scale line of the centrifuge tube. After centrifuging the culture solution (16,050 g, 5 min), the supernatant and the bacterial precipitation were collected, respectively. The content of Fe(II) in the supernatant was determined according to the method in the ferrous ion content assay kit (BL1147B, Biosharp). To quantify the amount of nucleic acid that leaked into the supernatant following the rupture of the bacterial cells during the culture process, the absorbance of the supernatant at 260 nm (A_260_) was determined using a multifunctional enzyme labeler (SpertraMax M2, Molecular Devices, San Jose, CA, USA) [32]. The intracellular reactive oxygen species (ROS) levels of the bacteria were measured using a ROS assay kit (BL714A, Biosharp). In brief, the bacterial precipitation (0.0020 g) and the H_2_DCFDA probe (300 μL, 10 μM) were placed into a 1.5 mL EP tube and then homogenized. The EP tube was incubated in a water bath at 37 °C for 30 min and shielded from light. The fluorescence intensity was finally measured using a multifunctional enzyme labeler, with excitation at 488 nm and emission at 525 nm. The total amount of free bacteria in the upper culture solution was monitored over time by measuring the optical density at 600 nm (OD_600_) of the culture solution at the 25 mL scale line of the centrifuge tube using a multifunctional enzyme marker. The pH of the culture solution was determined using a pH meter.

After determining the aforementioned indexes, the remaining culture solution was centrifuged (16,639 g, 8 min), and the supernatant was collected. The content of TFe in the supernatant was measured using a flame atomic absorption spectrometer. The content of lactic acid in the supernatant was detected using a high-performance liquid chromatograph (HPLC, 1260 Infinity II, Agilent, San Jose, CA, USA). The HPLC conditions were as follows: a Welch Ultimate AQ-C18 column (4.6 mm × 10 mm, 5 μm). The column temperature was 35 °C. The mobile phase was 97.5% phosphoric acid (0.1%) and 2.5% acetonitrile, and its flow rate was 1 mL/min. The detection wavelength was 210 nm.

## 3. Results

### 3.1. The Physicochemical Properties of Pit Mud and Yellow Soil

The results on the physicochemical properties of the pit mud with different fermentation pit ages and the yellow soil are displayed in Table 1. The physicochemical properties of the pit mud underwent significant alterations with an increase in fermentation pit age. The pH of the yellow soil was weakly acidic at 6.17, while the pH of the 2-year pit mud significantly decreased to 4.08 and that of the 100-year pit mud gradually increased to 6.58. The MC of the yellow soil was the lowest, at 7.72%, while the MC of pit mud significantly increased from 23.51% in the 2-year pit mud to 43.00% in the 100-year pit mud. In terms of iron-related indexes, the contents of TFe, Fe(III), Fe_d_, and Fe_c_ decreased significantly from the yellow soil to the 100-year pit mud, while the contents of Fe(II), Fe_o_, and Fe(II)/Fe(III) increased significantly. This evidence showed that the content and valence state of iron and the morphology of iron-bearing minerals changed significantly during aging of the pit mud.

### 3.2. The Composition and Structural Characteristics of the Prokaryotic Communities

After splicing and quality control of the original data, the number of prokaryotic sequences obtained from each sample exceeded 60,000. The processed prokaryotic data yielded effective sequences ranging from 65,637 to 113,741 with an average length of 409.12 bp per sample. Prokaryotic taxa encompassed a total of 50 phyla and 655 genera. The prokaryotic sampling coverage equaled or exceeded 99.7%, indicating that the identified sequences represented almost all of the prokaryotic sequences present in the sample.

The richness and diversity of the microorganisms in the yellow soil and pit mud were evaluated using the α diversity indexes. Table 2 indicates that the observed ASVs and Chao1 and Shannon indexes of the 2-year and 40-year pit mud were not significantly different, yet the values for both were significantly lower than those for the 100-year pit mud. Additionally, the Simpson indexes of the pit mud samples showed a significant increase with fermentation pit age. The four α diversity indexes of the yellow soil were significantly higher than those of the 2-year and 40-year pit mud. Moreover, the observed ASVs and Shannon and Chao1 indexes of the yellow soil were not significantly different from those of the 100-year pit mud, but the Simpson index of the yellow soil was significantly higher than that of the 100-year pit mud sample.

**Figure 1:**
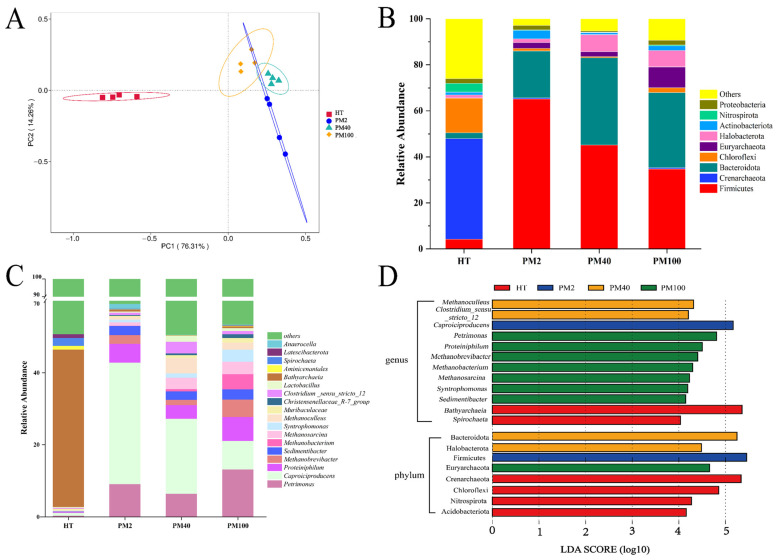
Prokaryotic community structure and composition of yellow soil and pit mud of different fermentation pit ages. (**A**) PCoA (based on Bray–Curtis distances). (**B**) Prokaryotic community composition at the phylum level (Top 9). (**C**) Prokaryotic community composition at the genus level (Top 18). (**D**) LEfSe analysis of prokaryotic communities at the phylum and genus levels.

A PCoA was adopted to evaluate the differences in the prokaryotic community structures among different groups. Figure 1A shows that the sample points for the yellow soil and pit mud were distributed on the left and right sides of the PC1 axis, respectively, indicating that the prokaryotic community structures of the yellow soil and pit mud samples were different. The analysis of similarity (ANOSIM) results further showed (Table S1) that there were significant differences in the prokaryotic community structures between the yellow soil and pit mud samples (*p* < 0.05).

The composition of prokaryotic communities at the phylum (Top 9) and genus levels (Top 18) in the yellow soil and pit mud of different fermentation pit ages was further investigated. ASVs with a relative abundance of less than 1% and an uncertain taxonomic status were classified as “others”. At the phylum level (Figure 1B), the three phyla with the largest proportions in yellow soil were Crenarchaeota (43.78%), Chloroflexi (15.10%), and Firmicutes (4.19%). In addition, Crenarchaeota, Chloroflexi, Acidobacteriota, and Nitrospirota were biomarkers in the yellow soil (Figure 1D). The four phyla with the highest relative abundance in pit mud were Firmicutes (34.71~65.10%), Bacteroidota (20.48~37.90%), Euryarchaeota (2.13~9.10%), and Halobacterota (1.67~7.45%), and these four phyla were biomarkers in the pit mud (Figure 1B,D).

At the genus level (Figure 1C), there were also significant differences in the composition of the prokaryotic communities between yellow soil and pit mud. The yellow soil was dominated by Bathyarchaeia (43.67%) and Spirochaeta (2.19%). The dominant genera in pit mud were *Clostridium_sensu_stricto_12* (0.68–3.28%), *Petrimonas* (6.40–13.18%), *Caproiciproducens* (7.88–33.75%), *Proteiniphilum* (3.77–6.61%), *Sedimentibacter* (2.35–2.88%), and *Syntrophomonas* (0.72–3.34%), and the relative abundance of bacteria in these genera gradually increased with fermentation pit age (Figure 1C,D). In addition, the relative abundance of Methanobrevibacter, Methanobacterium, Methanosarcina, and Methanoculleus increased with fermentation pit age, and these four Archaea genera constituted biomarkers in the 40-year and 100-year pit mud (Figure 1C,D). Notably, the relative abundance of *Lactobacillus* in the 2-year pit mud was low (0.33%), which did not appear to align with the typical abundance characteristics of *Lactobacillus* in young pit mud. Functional predictions of the prokaryotic communities showed that (Table S3) as the fermentation pit age increased, the functions of methanogenesis, hydrogenotrophic methanogenesis, and methanogenesis through CO_2_ reduction by H_2_ were significantly up-regulated, and the functions of methylotrophy and methanogenesis through the reduction of methyl compounds by H_2_ were significantly down-regulated (*p* < 0.05). This finding suggested that hydrogenotrophic methanogens in pit mud were the main methanogens.

### 3.3. The Correlation Analysis Between the Prokaryotic Communities and Environmental Factors

**Figure 2:**
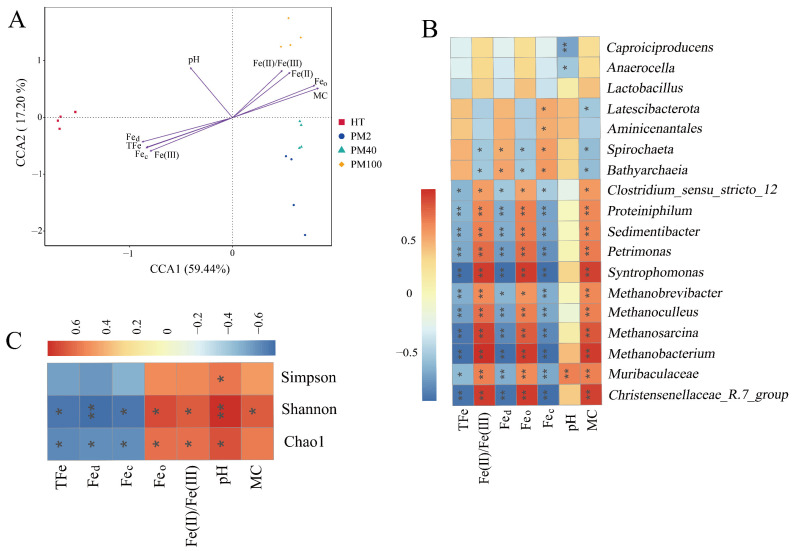
Correlation analysis between prokaryotic communities and environmental factors. (**A**) CCA. (**B**) Spearman’s rank correlation analysis of prokaryotic community genera (Top 18) and environmental factors. (**C**) Spearman’s rank correlation analysis of α diversity indexes of prokaryotic communities and environmental factors. * indicates *p* < 0.05. ** indicates *p* < 0.01.

A CCA was used to explore the important environmental factors affecting the distribution of prokaryotic communities at the genus level. Figure 2A demonstrates that the CCA1 and CCA2 axes together explained 76.64% of the variation in prokaryotic community composition, indicating a remarkable relationship between environmental factors and prokaryotic community structure. The results of using the envfit function further showed (Table S2) that all environmental factors had significant effects on the prokaryotic community distribution, among which TFe exerted the most significant effect on the prokaryotic community distribution (R^2^ = 0.979, *p* < 0.001). The Spearman’s rank correlation analysis was used to study the correlation between TFe, MC, Fe_c_, Fe_o_, Fe_d_, pH, Fe(II)/Fe(III), and prokaryotic genera (Top 18). Figure 2B indicates that several important functional microorganisms in pit mud, such as *Clostridium_sensu_stricto_12*, *Proteiniphilum*, *Sedimentibacter*, *Syntrophomonas*, *Petrimonas*, *Methanobrevibacter*, *Methanobacterium*, *Methanosarcina*, and *Methanoculleus*, were significantly negatively correlated with TFe, Fe_d_, and Fe_c_ but significantly positively correlated with Fe(II)/Fe(III), Fe_o_, and MC. It is worth noting that *Lactobacillus* showed negative correlations with TFe, Fe_d_, and Fe_c_ (though not significantly), which implied that the relative abundance of *Lactobacillus* at the community level might be influenced by the content of iron-bearing minerals. Additionally, as shown in Figure 2C, an analysis of the results of the Spearman’s rank correlation between the environmental factors and α diversity indexes of the prokaryotic communities in pit mud revealed that the Shannon index and the Chao1 index were significantly negatively correlated with TFe, Fe_d_, and Fe_c_ and positively correlated with Fe_o_, Fe(II)/Fe(III), and pH (*p* < 0.05). pH had a significant positive correlation with the Simpson index, while the MC only exhibited a significant positive correlation with the Shannon index. In general, changes in the iron-related indexes, pH, and MC remarkably affected the richness and diversity of the prokaryotic communities in the pit mud.

### 3.4. The Effects of Goethite on the Growth and Metabolism of Strain JN01

**Figure 3:**
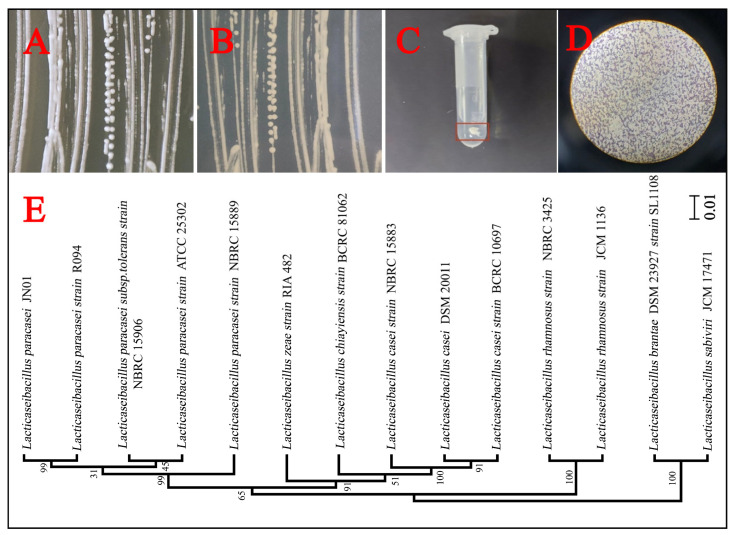
The isolation and identification of *Lacticaseibacillus paracasei* JN01. Colony characterization of strain JN01 on the front (**A**) and back (**B**) of the solid MRS medium. (**C**) Negative result in the catalase test: no bubbles. (**D**) Positive result in the Gram stain test: purple. (**E**) A phylogenetic tree of strain JN01 based on the 16S rDNA sequence (99% homology with *Lacticaseibacillus paracasei* strain R094). The scale bar of the phylogenetic tree is 0.01.

**Figure 4:**
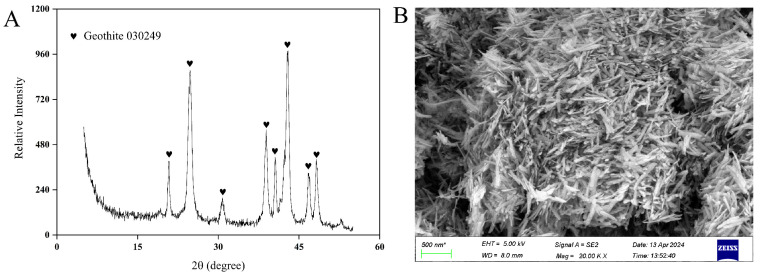
Characterization of goethite. (**A**) XRD and (**B**) SEM.

A total of four strains of *Lacticaseibacillus paracasei* were screened. Strain JN01 (with NCBI accession number SUB15087239), which exhibited the strongest activity and fastest growth, was selected for subsequent experiments. The colony morphology of strain JN01 was circular, with a white front and a yellow back (Figure 3A,B). The catalase test result was negative (no bubbles, Figure 3C), and the Gram staining result was positive (purple, Figure 3D). Strain JN01 and *Lacticaseibacillus paracasei* strain R094 showed the highest (99%) homology (Figure 3E). The XRD results for goethite (ICDD PDF File No. 03-0249) showed that all of the diffraction peaks were strong and sharp, suggesting that the goethite prepared in this study was well crystallized (Figure 4A). The SEM results indicated that the length of goethite was about 500 nm, and it exhibited an elongated, acicular morphology (Figure 4B).

**Figure 5:**
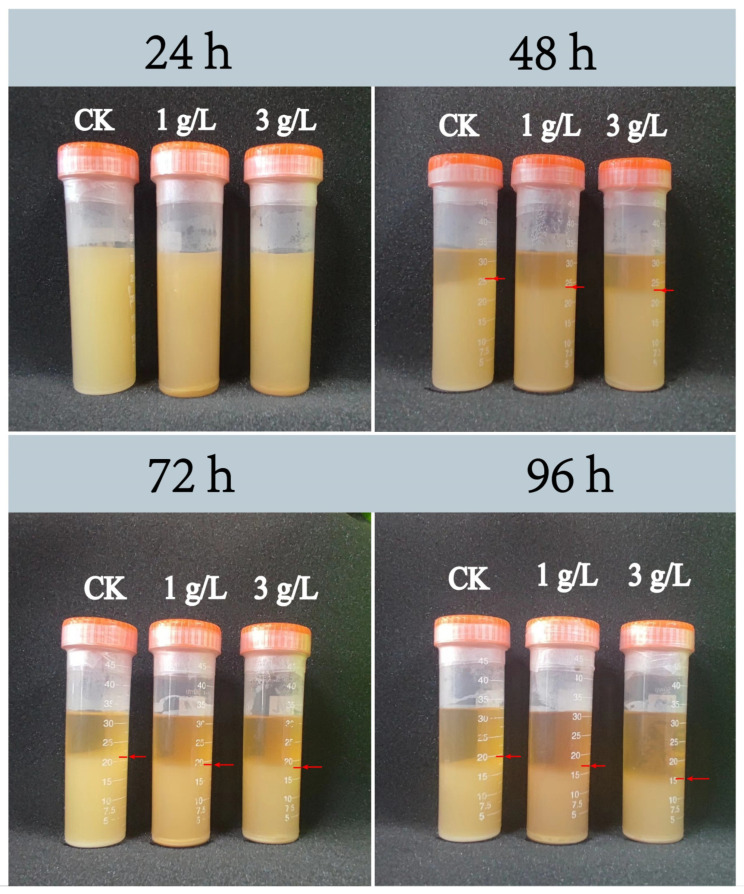
Deposition height of *Lacticaseibacillus paracasei* JN01 in CK, 1 g/L, and 3 g/L samples under static culture conditions.

The negative correlation between *Lactobacillus* and the iron-bearing mineral Fe_c_ was supposed as the reason for the unusually low abundance of *Lactobacillus* in the 2-year pit mud (Figure 2B). It was impractical to add Fe_c_, such as goethite (a typical representative of Fe_c_), in situ to the pit mud in Luzhou Laojiao distillery to observe the changes in the abundance of *Lactobacillus*. Therefore, this study investigated the effects of goethite on the growth and metabolism of strain JN01 further at the pure culture level in the laboratory, which intended to explain the unusually low abundance of *Lactobacillus* in the 2-year pit mud. As shown in Figure 5, when incubated for 24 h, the bacterial culture solutions of 1 g/L and 3 g/L and the CK were relatively cloudy, and there was no obvious difference visible to the naked eye. As the incubation time gradually extended to 48 h, 72 h, and 96 h, more bacteria in the culture solution settled at the bottom of the centrifuge tube, and the culture solution was stratified accordingly. This possibly could be attributed to the fact that the anaerobic environment at the bottom of the centrifuge tube was more suitable for the growth of strain JN01. The culture solution contained free bacteria in the upper layer and deposited bacterial cells in the lower layer. As shown by the red arrow in Figure 5, the height of bacterial deposition was CK > 1 g/L > 3 g/L.

**Figure 6:**
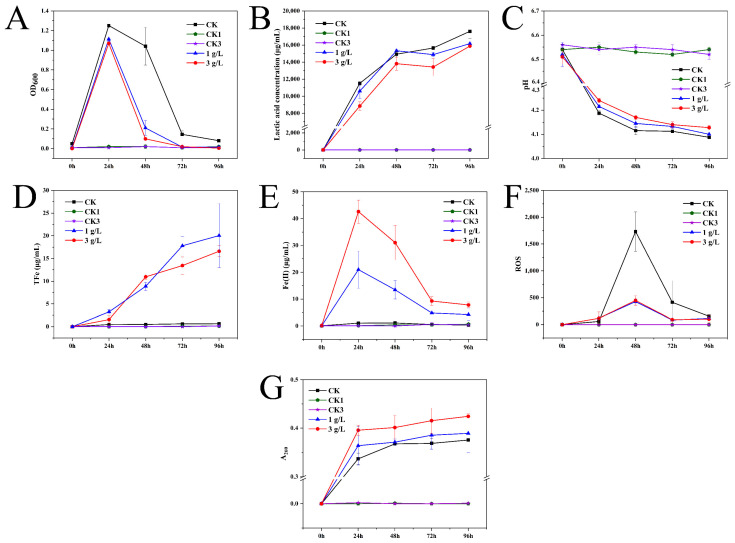
Physicochemical indexes of culture medium. (**A**) OD_600_, (**B**) lactic acid concentration, (**C**) pH, (**D**) total iron, (**E**) Fe(II), (**F**) ROS, and (**G**) A_260_.

To reflect the dynamic changes in the bacterial settlement with incubation time, 200 μL of culture solution was collected at the 25 mL scale line of the centrifuge tube, and the OD_600_ of the culture solution was measured. The OD_600_ decreased over time as a result of the continuous sedimentation of bacteria to the bottom of the centrifuge tube. As evidenced by CK > 1 g/L > 3 g/L, the differences in OD_600_ indicated that the growth rate of strain JN01 may be inhibited by goethite in a concentration-dependent manner (Figure 6A). As lactic acid is the main metabolite of *Lacticaseibacillus*, its secretion and pH in the culture solution can be used to monitor the physiological and metabolic status of strain JN01. As shown in Figure 6B,C, the concentration of lactic acid in the CK culture solution was the highest, and correspondingly, the pH of the CK solution was the lowest, while the lactic acid concentration in the specimens in the 1 g/L group was higher than that of those in the 3 g/L group, and the corresponding pH at 1 g/L was lower than that at 3 g/L. This finding indicated that the addition of goethite could reduce the secretion of lactic acid by strain JN01. Additionally, OD_600_, lactic acid concentration, and pH in the CK1 and CK2 groups remained stable with culture time (Figure 6A–C).

To investigate the effect of the metabolic activity of strain JN01 on goethite, the concentrations of TFe and Fe(II) in the culture solution were determined. The result in Figure 6D demonstrates that in general, the TFe concentration at 1 g/L was higher than that in the specimens in the 3 g/L group, indicating a lower weathering effect of the bacteria at 3 g/L on the goethite compared to that of the bacteria at 1 g/L. As shown in Figure 6E, the Fe(II) concentration at 3 g/L and 1 g/L gradually decreased from 24 h to 96 h, implying that the Fe(II) in the culture solution might have gradually entered into the bacterial cells. Additionally, the Fe(II) concentration in the culture solution was such that 3 g/L > 1 g/L, suggesting a potentially higher uptake of Fe(II) by the bacterial cells at 3 g/L. In addition, goethite was still present at 96 h, indicating that it had not been completely weathered by strain JN01. Since the CK1 and CK2 groups were not inoculated with bacteria, the TFe and Fe(II) concentrations in these groups remained almost unchanged (Figure 6D,E).

The intracellular ROS levels were quantified to analyze the intracellular response of strain JN01 under goethite stress. Figure 6F shows that at 24 h, the intracellular ROS levels of strain JN01 in 3 g/L and 1 g/L were higher than those in the CK solution, indicating that the addition of goethite could stimulate the production of bacterial intracellular ROS. From 48 h to 96 h, the intracellular ROS levels in the CK, 1 g/L, and 3 g/L samples gradually decreased, which may have been due to rupture of the bacterial cells, resulting in the release of bacterial intracellular ROS into the solution. Moreover, from 24 h to 96 h, the intracellular ROS levels in the CK solution were higher than those in the specimens from the 3 g/L and 1 g/L groups, suggesting that goethite may expedite bacterial death and cellular rupture, consequently facilitating the accelerated release of intracellular ROS into the solution. The quantity of nucleic acid in the culture solution was further assessed to characterize the extent of bacterial rupture during cultivation. The results in Figure 6G show that from 24 h to 96 h, the amount of nucleic acids in the 3 g/L, 1 g/L, and CK culture solutions gradually increased. Notably, the concentration of the nucleic acids consistently followed the order of 3 g/L > 1 g/L > CK, indicating that the addition of goethite enhanced bacterial rupture, with a higher concentration of goethite resulting in greater bacterial rupture. No measurable ROS or nucleic acid levels were detected in the CK1 and CK2 groups because they were not inoculated with bacteria (Figure 6F,G).

## 4. Discussion

### 4.1. Differences in the Prokaryotic Community Composition Between Pit Mud and Yellow Soil

Since yellow soil is an essential raw material for the preparation of pit mud, understanding the differences in the prokaryotic communities between yellow soil and pit mud facilitates greater comprehension of the evolution process of the microbial communities in pit mud. Yellow soil, due to its frequent exposure to air and sunlight and deficiency in nutrients, exhibited significant enrichment in aerobic bacteria (such as Acidobacteriota and Nitrospirota) and phototrophic bacteria (such as Chloroflexi) [33,34,35] (Figure 1D). When yellow soil was used as the raw material for pit mud production and subsequently placed in a fermentation pit, the anaerobic and light-deprived fermentation environment within the fermentation pit proved unfavorable for the survival of these bacteria, leading to a significant decrease in their relative abundance within the pit mud. Therefore, the α diversity indexes of the 2-year and 40-year pit mud samples were lower than that of the yellow soil (Table 2). However, the α diversity indexes of the 100-year pit mud samples continued to increase. Previous studies found that a near-neutral pH environment was generally more favorable for the growth and metabolism of most prokaryotic organisms [36]. Moreover, the excessively high TFe content in pit mud was detrimental to the survival of the microorganisms inhabiting it [37]. Iron is a trace element that is very important for life activities. If its concentration is too high, it will inhibit the life activities of organisms. For this reason, a further reduction in the TFe content and the increase in pH in the 100-year pit mud samples optimized the physicochemical environment of the pit mud, thereby promoting an increase in the α diversity of the 100-year pit mud. In addition, some of the microorganisms in fermented grains may be transferred to the pit mud along with yellow water during the fermentation process, thereby promoting an increase in α diversity in the 100-year pit mud [38].

A variety of dominant fermentation microorganisms, such as *Petrimonas*, *Syntrophomonas*, and *Clostridium*, that inhabit pit mud are widely regarded as important contributors to high-quality liquor [1,9]. *Petrimonas* can decompose organic matter and produce acetic acid, propionic acid, etc. [39]. *Syntrophomonas* can degrade C_4_–C_8_ fatty acids into acetic acid and propionic acid [40]. *Sedimentibacter* can ferment amino acids into acetic acid and butyric acid [41]. *Clostridium* is an important contributor to caproic acid in pit mud, and caproic acid is an important precursor substance for the synthesis of ethyl caproate, a typical flavor substance in strong-flavor *baijiu* [7,8]. In our previous study, the content of aromatic compounds in the dissolved organic matter of the pit mud increased with the aging of the pit mud [10], which might be attributed to the increased abundance of the aforementioned fermentative microorganisms in pit mud.

In the anaerobic environment of the pit mud, H_2_ is generated through the metabolic activities of fermentation microorganisms. However, excessive accumulation of H_2_ can result in hydrogen inhibition, which is detrimental to the survival of fermentation microorganisms [42]. An increased relative abundance of hydrogenotrophic methanogens is considered to be beneficial to the aging of pit mud [3]. This is because hydrogenotrophic methanogens and fermentation bacteria can alleviate hydrogen inhibition during fermentation through interspecies hydrogen transfer, thereby promoting the production of short-chain fatty acids [43,44]. Moreover, *Methanoculleus* and *Methanosarcina* contain a large number of genes encoding glycosyltransferases, which are crucial for the breakdown and transformation of carbohydrate substances during the fermentation process of strong-flavor *baijiu* [1]. Therefore, hydrogenotrophic methanogens can also indirectly affect the production of alcohol and the taste of the final product. In this study, the relative abundance of *Methanobrevibacter*, *Methanobacterium*, *Methanosarcina*, and *Methanoculleus* increased with the fermentation pit age (Figure 1C). These methanogens are hydrogenotrophic methanogens capable of utilizing H_2_ and CO_2_ for the production of CH_4_ [8]. Therefore, an increased relative abundance of hydrogenotrophic methanogens is also a crucial factor contributing to “high-quality liquor produced in an old fermentation pit” [8,45].

### 4.2. The Effects of Iron-Bearing Minerals in Pit Mud on the Prokaryotic Community Structure

Environmental factors play an important role in shaping the structure and composition of prokaryotic communities in pit mud [46]. Previous studies have found that an excessively high TFe content in pit mud can weaken the water-holding and water-permeability of the pit mud and harden its surface [37], which could potentially account for the negative correlation between TFe and MC shown in Figure 2A. Furthermore, with an increase in fermentation pit age, the TFe content in pit mud showed a gradually decreasing trend [11,47]. Nevertheless, these studies failed to set out to assess the correlation between TFe and the prokaryotic communities in pit mud. Furthermore, given that iron-bearing minerals serve as a crucial source of iron in pit mud, it is imperative to investigate the relationship between iron-bearing minerals and prokaryotic communities in pit mud.

Hydrogenotrophic methanogens are key to maintaining the stability of the pit mud ecosystem [3]. The presence of iron-bearing minerals can affect the development of communities of methanogens [48]. It was reported that the addition of ferrihydrite (an amorphous iron (hydr)oxide) decreased the relative abundance of hydrogenotrophic methanogens in the deep sediments of the South China Sea and increased the relative abundance of methylotrophic methanogens [18]. The results in Table 1 show that the content of amorphous iron (hydr)oxides increased significantly with fermentation pit age, which may be unfavorable to the enrichment of hydrogenotrophic methanogens. Moreover, the contents of Fe(II) and Fe(III) can exert an important influence on the activity of methanogens. A high Fe(III) content (e.g., 0.9 mmol/g of volatile suspended solids) was found to have a severe inhibitory effect on the growth of methanogens [49]. It was discovered that Fe(II) can precipitate CO_2_ to form FeCO_3_, thereby suppressing the utilization of CO_2_ by methanogens, impeding their growth and reproduction [50]. In the present study, the increasing Fe(II) content (from 0.46 g/kg in the yellow soil to 3.91 g/kg in the 100-year pit mud) with fermentation pit age may have reacted with the CO_2_ produced by the microbial metabolism to form FeCO_3_, thus inhibiting the growth of hydrogenotrophic methanogens by reducing the amount of CO_2_ ingested by hydrogenotrophic methanogens. In addition, an increase in MC has been demonstrated to be conducive to creating a reducing environment, accelerating the reduction and dissolution of crystalline iron minerals, and promoting the accumulation of Fe(II) [51]. Therefore, the high content of TFe and the continuous increase in amorphous iron (hydr)oxides and Fe(II) in young pit mud may be detrimental to a rapid enrichment in hydrogenotrophic methanogens. Methanogens and fermentation bacteria have an intermetabolic relationship, and methanogens play a crucial role in maintaining the stability of the pit mud ecosystem. Therefore, amorphous iron (hydr)oxides may indirectly hinder the growth and enrichment of fermentation bacteria by slowing the enrichment in methanogens, thus delaying the aging of pit mud.

The richness and diversity of prokaryotic communities in pit mud are closely related to the physicochemical environment. Figure 2B,C show that Fe_c_ was significantly negatively correlated with certain fermentation bacteria (e.g., *Proteiniphilum*, *Sedimentibacter*, *Syntrophomonas*, and *Petrimonas*) and the Chao1 and Shannon indexes of the prokaryotic communities, indicating that the diversity of the prokaryotic communities in pit mud may be significantly influenced by the content of Fe_c_.

### 4.3. The Mechanism of Goethite’s Effect on Strain JN01’s Physiology and Metabolism

Notably, the relative abundance of *Lactobacillus* in the 2-year pit mud was too low (Figure 1C), which seems to be inconsistent with the dominance of *Lactobacillus* in young pit mud observed in previous studies [52,53]. The result in Figure 2B shows that Fe_c_ was negatively correlated with the relative abundance of *Lactobacillus*. Compared with aged pit mud, Fe_c_, such as goethite, was higher in young pit mud [10]. Therefore, the content of Fe_c_ may be an important factor affecting the abundance of *Lactobacillus* in pit mud. In this study, goethite inhibited the growth and metabolism of strain JN01 in a concentration-dependent manner under pure culture conditions in a laboratory. Compared with CK solution, the addition of goethite decreased the deposition height of the bacteria, OD_600_, and lactic acid secretion (Figure 5 and Figure 6A,B) in the 1 g/L and 3 g/L samples. Previous studies have proven that iron-bearing minerals can affect the physiological status of bacteria through their morphology, surface physical properties, and chemical composition [16,54]. Nano-sized goethite was found to be able to penetrate the outer membrane and the peptidoglycan layer of *Shewanella putrefaciens* (the possibility of centripetal shear force pushing nano-sized goethite into the bacteria was ruled out in this study), thus affecting the normal physiological activity of the bacteria [55]. A strong and close association between *Escherichia coli* O157:H7 and acicular goethite was revealed to cause serious damage to the bacterial cell membrane and reduce the bacterial activity [30]. In this study, the contact between nano-sized acicular goethite and strain JN01 under static culture conditions may have damaged the integrity of the cell membranes of strain JN01. Furthermore, pH affected the amount of surface charge in the goethite. The zero charge point on the surface of goethite ranged between a pH of 7.4 and 8.5, and the lower pH was, the more positive the charge on the surface of the goethite was [56,57]. As the incubation time was prolonged, the amount of lactic acid secreted by strain JN01 increased, causing the pH value of the culture solution to drop below 6.5 (Figure 6C). Therefore, the surface of the goethite consistently exhibited a positive charge, which intensified as the pH decreased. The surface of *Lacticaseibacillus* typically exhibited a negative charge [58,59]. As a result, the electrostatic attraction between the bacteria and the goethite increased with incubation time, leading to closer interaction between them. Therefore, 3 g/L, which contained more nano-sized acicular goethite than 1 g/L, had a stronger effect on the destruction of the cell membranes and the physiological activity of strain JN01. In addition, previous studies found that Fe(II) and Fe(III) may have a toxic effect on bacterial growth. It is believed that Fe(II) dissolved from iron-bearing clay can enter bacterial cells and be oxidized, resulting in the toxic precipitation of Fe(III) and destructive hydroxyl radicals, which ultimately inactivate bacteria [17]. Under anaerobic conditions, where the oxidative damage caused by Fe(II)’s aerobic oxidation was absent, Fe(III) was still found to reduce the activity of *Salmonella* pmrA mutants and damage their bacterial outer membranes [60]. In this study, the content of Fe(II) in the 1 g/L and 3 g/L samples decreased gradually over time, which may have been because Fe(II) entered strain JN01’s cells. The progressive accumulation of Fe(II) in bacterial cells may stimulate the production of intracellular ROS in strain JN01 (Figure 6F), causing bacterial oxidative stress damage and eventually accelerating cell rupture and the release of intracellular ROS and nucleic acids. Therefore, the low relative abundance of *Lactobacillus* in the 2-year pit mud may have been related to the high content of Fe_c_ in the 2-year pit mud.

The aforementioned results inspire us to consider that the content of iron-bearing minerals may be an important environmental factor affecting the diversity of the prokaryotic community in pit mud. Moreover, yellow soil, as an essential raw material for the production of pit mud in Luzhou Laojiao distillery, contributes most of the iron in the subsequent preparation of pit mud. The TFe value of around 31 g/Kg from this study may represent the typical level of TFe in the yellow soil used in the pit mud production practice of Luzhou Laojiao distillery. The contents of TFe and iron-bearing minerals in yellow soil may affect the quality of pit mud to some extent. Therefore, it is necessary to pay attention to the TFe and iron-bearing mineral contents in the yellow soil in advance. For example, adopting certain biochemical strategies in advance to reduce the TFe in the yellow soil to below 20 g/Kg (which represents the TFe level of pit mud around 40 years old) may help with preparing higher-quality pit mud and strong-flavor *baijiu*.

## 5. Conclusions

The contrasting physicochemical environments of yellow soil and pit mud led to a significant difference in the prokaryotic community composition between them. The relative abundance of several dominant functional bacteria and hydrogenotrophic methanogens in the pit mud increased gradually with increasing fermentation pit age. Under laboratory conditions, goethite (typical representative of Fe_c_) can decrease the physiological metabolic activity of *Lacticaseibacillus paracasei* JN01 in a concentration-dependent manner. TFe and Fe_c_ showed significant negative correlations with both the Chao1 and Shannon indexes and key microorganisms such as *Clostridium_sensu_stricto_12*, *Sedimentibacter*, *Petrimonas*, *Syntrophomonas*, and hydrogenotrophic methanogens. Fe_o_ may not be conducive to enrichment in hydrogenotrophic methanogens in pit mud. Choosing the appropriate yellow soil with a low iron content will help enhance the quality of the prepared pit mud. Taken collectively, the contents of TFe and iron-bearing minerals may be important factors influencing the community structure of prokaryotic microorganisms in the pit mud of Luzhou Laojiao distillery. This study, by exploring the interactions between the prokaryotic microorganisms and iron-bearing minerals in pit mud, offers a new perspective for understanding the aging process of pit mud.

## Figures and Tables

**Table 1:** Physicochemical properties of yellow soil and pit mud with different fermentation pit ages.

Sample	HT	PM2	PM40	PM100
pH	6.17 ± 0.03 b	4.08 ± 0.03 d	5.06 ± 0.19 c	6.58 ± 0.13 a
MC (%)	7.72 ± 0.40 d	23.51 ± 1.31 c	32.41 ± 2.70 b	43.00 ± 2.21 a
TFe (g/kg)	31.57 ± 0.94 a	24.01 ± 1.36 b	19.98 ± 0.50 c	15.20 ± 0.24 d
Fe(II) (g/kg)	0.46 ± 0.03 d	0.75 ± 0.02 c	1.98 ± 0.13 b	3.91 ± 0.24 a
Fe(III) (g/kg)	31.11 ± 0.97 a	23.25 ± 1.36 b	17.99 ± 0.39 c	11.30 ± 0.40 d
Fe(II)/Fe(III)	0.01 ± 0.00 c	0.03 ± 0.00 c	0.11 ± 0.01 b	0.35 ± 0.03 a
Fe_d_ (g/kg)	12.26 ± 0.40 a	10.47 ± 0.28 b	9.34 ± 0.36 c	8.81 ± 0.06 d
Fe_o_ (g/kg)	1.55 ± 0.19 d	3.69 ± 0.03 c	6.12 ± 0.10 b	7.50 ± 0.42 a
Fe_c_ (g/kg)	10.72 ± 0.49 a	6.78 ± 0.26 b	3.21 ± 0.44 c	1.30 ± 0.41 d

Note: MC, moisture content; TFe, total iron; Fe(II)/Fe(III), the ratio of bivalent iron to trivalent iron; Fe_d_, free iron minerals; Fe_o_, amorphous iron (hydr)oxides; Fe_c_, crystalline iron minerals. HT, yellow soil. PM2, PM40, and PM100 refer to 2-year, 40-year, and 100-year pit mud, respectively. Results in the table are expressed as the mean ± standard deviation (*n* = 4). Different lowercase letters on the same line (a–d) indicate that there are significant differences between the physicochemical properties of different samples (*p* < 0.05).

**Table 2:** The α diversity indexes of yellow soil and pit mud of different fermentation pit ages.

Sample	Observed ASVs	Chao1	Shannon	Simpson
HT	1020.75 ± 140.07 a	1031.87 ± 159.39 a	7.67 ± 0.34 a	0.99 ± 0.00 a
PM2	637.00 ± 204.52 b	657.80 ± 200.99 b	5.71 ± 0.97 b	0.93 ± 0.04 cd
PM40	616.25 ± 93.68 b	632.35 ± 102.13 b	6.22 ± 0.46 b	0.96 ± 0.02 bc
PM100	1110.50 ± 164.80 a	1153.26 ± 159.39 a	7.10 ± 0.46 a	0.97 ± 0.01 b

Note: Results in the table are expressed as the mean ± standard deviation (*n* = 4). Different lowercase letters on the same line (a–d) indicate that there are significant differences between the α diversity indexes of different samples (*p* < 0.05).

## Data Availability

The original contributions presented in the study are included in the article/Supplementary Files, further inquiries can be directed to the corresponding author.

## Supplementary Materials

The following supporting information can be downloaded at: https://www.mdpi.com/article/10.3390/foods14111883/s1, Table S1: ANOSIM test results of prokaryotic communities structure; Table S2: Envfit function test results; Table S3: Functional notes of prokaryotic communities (Top10).
