# Supplementary material for: The Effects of Iron-Bearing Minerals on the Community Diversity and Physiological Activity of Prokaryotic Microorganisms in Pit Mud Used for Strong-Flavor baijiu Production
Source: Foods. 2025 May 26;14(11):1883. doi: 10.3390/foods14111883 (PMC12154080; doi:10.3390/foods14111883)
Supplement: Supplementary file 1 [file foods-14-01883-s001.zip › foods-3639893-supplementary.pdf]

Table S1 ANOSIM test results of prokaryotic communities structure

Group	R-value	*p*
HT-PM2	1.00	0.033
HT-PM40	1.00	0.038
HT-PM100	1.00	0.032
PM2-PM40	1.00	0.023
PM2-PM100	1.00	0.030
PM40-PM100	0.99	0.028

Table S2 Envfit function test results

	CCA1	CCA2	r^2	*p*
TFe	-0.8567	-0.5159	0.9790	0.0005
Fe(III)	-0.8193	-0.5734	0.9748	0.0005
MC	0.8596	0.5109	0.9600	0.0005
Fe_c	-0.8600	-0.5103	0.9596	0.0005
Fe_o	0.8278	0.5610	0.9512	0.0005
Fe_d	-0.9074	-0.4204	0.9490	0.0005
Fe(II)	0.5952	0.8036	0.9209	0.0005
pH	-0.4372	0.8993	0.9050	0.0005
Fe(II)/Fe(III)	0.5235	0.8520	0.8943	0.0020

Table S3 Functional notes of prokaryotic communities (Top10)

Feature%	HT	PM2	PM40	PM100
Methanogenesis	2.33b	3.75b	7.92a	10.10a
Hydrogenotrophic methanogenesis	2.30b	3.75b	7.92a	10.10a
Methanogenesis by CO_2 reduction with H_2	0.36c	3.43bc	6.96ab	9.31a
Dark hydrogen oxidation	0.13c	2.88b	3.85b	8.03a
Fermentation	2.79a	3.57a	4.36a	3.98a
Methylotrophy	2.05a	0.33b	0.97ab	0.81b
Methanogenesis by reduction of methyl compounds with H_2	1.94a	0.33b	0.97ab	0.78ab
Aerobic chemoheterotrophy	1.06a	1.77a	0.54a	1.16a
Chemoheterotrophy	5.84a	5.53a	5.70a	5.86a
Others	2.45a	2.31a	1.00a	1.20a
